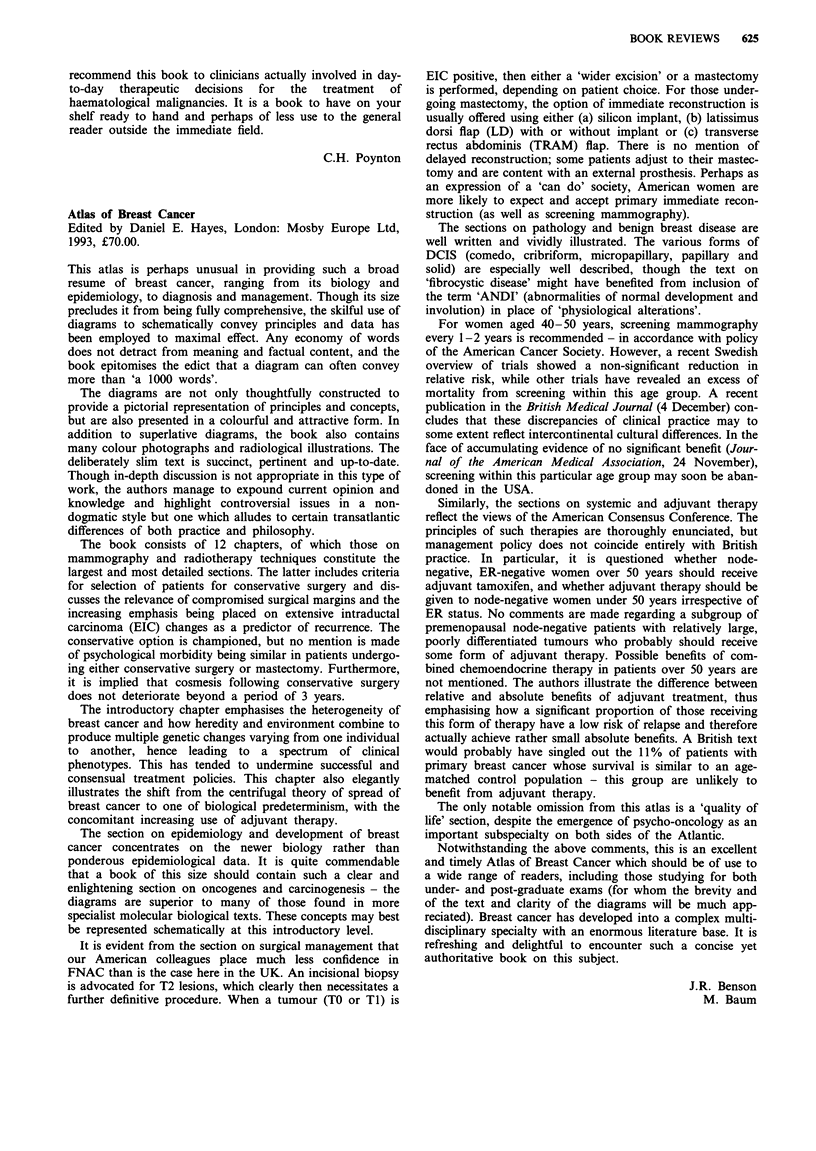# Atlas of Breast Cancer

**Published:** 1994-03

**Authors:** J.R. Benson, M. Baum


**Atlas of Breast Cancer**
Edited by Daniel E. Hayes, London: Mosby Europe Ltd, 1993, £70.00.

This atlas is perhaps unusual in providing such a broad resume of breast cancer, ranging from its biology and epidemiology, to diagnosis and management. Though its size precludes it from being fully comprehensive, the skilful use of diagrams to schematically convey principles and data has been employed to maximal effect. Any economy of words does not detract from meaning and factual content, and the book epitomises the edict that a diagram can often convey more than 'a 1000 words'.

The diagrams are not only thoughtfully constructed to provide a pictorial representation of principles and concepts, but are also presented in a colourful and attractive form. In addition to superlative diagrams, the book also contains many colour photographs and radiological illustrations. The deliberately slim text is succinct, pertinent and up-to-date. Though in-depth discussion is not appropriate in this type of work, the authors manage to expound current opinion and knowledge and highlight controversial issues in a non-dogmatic style but one which alludes to certain transatlantic differences of both practice and philosophy.

The book consists of 12 chapters, of which those on mammography and radiotherapy techniques constitute the largest and most detailed sections. The latter includes criteria for selection of patients for conservative surgery and discusses the relevance of compromised surgical margins and the increasing emphasis being placed on extensive intraductal carcinoma (EIC) changes as a predictor of recurrence. The conservative option is championed, but no mention is made of psychological morbidity being similar in patients undergoing either conservative surgery or mastectomy. Furthermore, it is implied that cosmesis following conservative surgery does not deteriorate beyond a period of 3 years.

The introductory chapter emphasises the heterogeneity of breast cancer and how heredity and environment combine to produce multiple genetic changes varying from one individual to another, hence leading to a spectrum of clinical phenotypes. This has tended to undermine successful and consensual treatment policies. This chapter also elegantly illustrates the shift from the centrifugal theory of spread of breast cancer to one of biological predeterminism, with the concomitant increasing use of adjuvant therapy.

The section on epidemiology and development of breast cancer concentrates on the newer biology rather than ponderous epidemiological data. It is quite commendable that a book of this size should contain such a clear and enlightening section on oncogenes and carcinogenesis – the diagrams are superior to many of those found in more specialist molecular biological texts. These concepts may best be represented schematically at this introductory level.

It is evident from the section on surgical management that our American colleagues place much less confidence in FNAC than is the case here in the UK. An incisional biopsy is advocated for T2 lesions, which clearly then necessitates a further definitive procedure. When a tumour (T0 or T1) is EIC positive, then either a 'wider excision' or a mastectomy is performed, depending on patient choice. For those undergoing mastectomy, the option of immediate reconstruction is usually offered using either (a) silicon implant, (b) latissimus dorsi flap (LD) with or without implant or (c) transverse rectus abdominis (TRAM) flap. There is no mention of delayed reconstruction; some patients adjust to their mastectomy and are content with an external prosthesis. Perhaps as an expression of a 'can do' society, American women are more likely to expect and accept primary immediate reconstruction (as well as screening mammography).

The sections on pathology and benign breast disease are well written and vividly illustrated. The various forms of DCIS (comedo, cribriform, micropapillary, papillary and solid) are especially well described, though the text on 'fibrocystic disease' might have benefited from inclusion of the term 'ANDI' (abnormalities of normal development and involution) in place of 'physiological alterations'.

For women aged 40–50 years, screening mammography every 1–2 years is recommended – in accordance with policy of the American Cancer Society. However, a recent Swedish overview of trials showed a non-significant reduction in relative risk, while other trials have revealed an excess of mortality from screening within this age group. A recent publication in the *British Medical Journal* (4 December) concludes that these discrepancies of clinical practice may to some extent reflect intercontinental cultural differences. In the face of accumulating evidence of no significant benefit (*Journal of the American Medical Association*, 24 November), screening within this particular age group may soon be abandoned in the USA.

Similarly, the sections on systemic and adjuvant therapy reflect the views of the American Consensus Conference. The principles of such therapies are thoroughly enunciated, but management policy does not coincide entirely with British practice. In particular, it is questioned whether node-negative, ER-negative women over 50 years should receive adjuvant tamoxifen, and whether adjuvant therapy should be given to node-negative women under 50 years irrespective of ER status. No comments are made regarding a subgroup of premenopausal node-negative patients with relatively large, poorly differentiated tumours who probably should receive some form of adjuvant therapy. Possible benefits of combined chemoendocrine therapy in patients over 50 years are not mentioned. The authors illustrate the difference between relative and absolute benefits of adjuvant treatment, thus emphasising how a significant proportion of those receiving this form of therapy have a low risk of relapse and therefore actually achieve rather small absolute benefits. A British text would probably have singled out the 11% of patients with primary breast cancer whose survival is similar to an age-matched control population – this group are unlikely to benefit from adjuvant therapy.

The only notable omission from this atlas is a 'quality of life' section, despite the emergence of psycho-oncology as an important subspecialty on both sides of the Atlantic.

Notwithstanding the above comments, this is an excellent and timely Atlas of Breast Cancer which should be of use to a wide range of readers, including those studying for both under- and post-graduate exams (for whom the brevity and of the text and clarity of the diagrams will be much appreciated). Breast cancer has developed into a complex multidisciplinary specialty with an enormous literature base. It is refreshing and delightful to encounter such a concise yet authoritative book on this subject.

J.R. Benson
M. Baum